# Computational Design of α-AsP/γ-AsP Vertical Two-Dimensional Homojunction for Photovoltaic Applications

**DOI:** 10.3390/nano12101662

**Published:** 2022-05-13

**Authors:** Yuliang Mao, Yuting Du, Zhipeng Huang, Guanhua Zhang, Jianmei Yuan

**Affiliations:** 1 Hunan Key Laboratory for Micro-Nano Energy Materials and Devices, School of Physics and Optoelectronics, Xiangtan University, Xiangtan 411105, China; 2 Hunan Key Laboratory for Computation and Simulation in Science and Engineering, School of Mathematics and Computational Science, Xiangtan University, Xiangtan 411105, China; 3 Hunan National Center for Applied Mathematics, Xiangtan 411105, China

**Keywords:** AsP, homojunction, photoelectric conversion efficiency, strain, first-principles

## Abstract

Based on first-principles calculations, we design a α-AsP/γ-AsP homojunction with minimum lattice distortion. It is found that the α-AsP/γ-AsP homojunction has an indirect bandgap with an intrinsic type-II band alignment. The proposed α-AsP/γ-AsP homojunction exhibits high optical absorption of $1.6\times 10^{6}{\;\mathrm{cm}}^{-1}$ along the zigzag direction. A high power conversion efficiency (PCE) of 21.08% is achieved in the designed α-AsP/γ-AsP homojunction, which implies it has potential applications in solar cells. Under 4% in-plane axial strain along the zigzag direction, a transition from indirect band gap to direct band gap is found in the α-AsP/γ-AsP homojunction. Moreover, the intrinsic type-II band alignment can be tuned to type-I band alignment under in-plane strain, which is crucial for its potential application in optical devices.

## 1. Introduction

The 2D nanosheet of group-Velement such as phosphorus has attracted broad attentions [1]. Phosphorene can be obtained by mechanical exfoliation of black phosphorus. Due to its high mobility and suitable band gap for photoelectric application, phosphorene is regarded as a promising semiconductor that can be used in nanodevices [2]. It was also reported that phosphorene has potential application in infrared detection [3]. As another new 2D nanosheet of group-Ⅴelement, arsenicene [4] was successfully synthesized in experiments [5,6]. It was reported that arsenicene has an indirect band gap of 2.49 eV. Under air humidity conditions, it was reported that phosphorus will greatly reduce its stability by oxidation and photo-induced degradation. During the preparation of arsenicene, arsenic trioxide is produced as an intermediate product. Since arsenic trioxide is highly toxic, it hinders the experimental study of arsenicence. It is thus intriguing to explore the possible arsenicence compound through material design [7,8].

Recently, phosphorus-arsenic alloy (As_x_P_1−x_) formed by phosphorene and arsine was successfully prepared in experiments [9,10]. Black arsenic phosphorus (b-AsP), which has a phase named α-AsP, exhibits potential optoelectronic applications due to its anisotropic optical absorption properties and good absorption properties in the mid-infrared region [9]. Based on arsenic-phosphorus, Zhou et al. [11] successfully fabricated field-effect transistors with anisotropic gate control ability and carrier mobility up to $10^{4}{\;\mathrm{cm}}^{2}V^{-1}s^{-1}$. Recently, we reported that the band gap and effective mass of α-AsP can be tuned by in-plane strain [12]. In the literature, five kinds of arsenic-phosphorus (AsP) allotropes were proposed, which were named α-, β-, γ-, δ-, and ε-AsP [13,14,15]. Xie et al. [16] reported that the five allotropes are stable by calculating their phonon spectrum.

It is reported that the optoelectronic properties of materials can be improved by constructing special heterojunctions. For example, the photocatalyst performance of the Zno/CuO heterojunction is excellent [17]. The light absorption and luminescence of semiconductors are directly affected by the absorption and recombination of excitons. It was reported that the InGaN/GaN [18] quantum wells of excitons have high luminous efficiency. In general, excitons are electron-hole pairs held together by Coulomb interaction. The further it spreads, the more sunlight can be absorbed. On the basis of viewpoint, a high-throughput computational screening of 1540 exciton solar cells composed by vertical heterostructures was explored [19]. In an experiment, Shojaei et al. [14] prepared the excitonic solar cells based on the design of AsP/GeSe 2D heterojunction with a relative high power conversion efficiency (PCE) of 16.0%. Recently, we reported that GeSe/SnSe heterojunction has a high PCE of 21.47% and a hole mobility of $6.42\times 10^{4}{\;\mathrm{cm}}^{2}V^{-1}s^{-1}\;$ [20]. For the band structure of heterojunction, it is generally believed that type-II band alignment means that the conduction band minimum (CBM) and the valence band maximum (VBM) of the heterojunction are contributed by two monolayer materials. In contrast, type-I band alignment means that the CBM and VBM of the heterojunction are only derived from one of the monolayer materials [21,22].

Although a large number of heterojunctions have been successfully reported, there are few reports on homojunctions. It was reported that the homojunction composed by different phases of MoTe_2_ was prepared [23,24]. Wu et al. [25] reported the successful preparation of the homojunction through the combination of 1T phase and 2H phase of transition metal chalcogenides, which provides a good solution for exploring the potential application of 2D Transition Metal Dichalcogenide (TMD) materials. This motivated us to design a α-AsP/γ-AsP homojunction, which has still not been reported.

In our study, we constructed a vertical homojunctions composed by the α-AsP monolayer and γ-AsP monolayer. We predicted their stability by considering different stacking orders. The detail of the band structure and charge transfer between the interlayer of the two AsP phases are analyzed. Our simulation indicates that the α-AsP/γ-AsP homojunction exhibits anisotropic characteristics and superior optical properties. We found that the band gap of the α-AsP/γ-AsP homojunction can be tuned by in-plane strain.

## 2. Computational Methods

To study the electronic properties of the α-AsP/γ-AsP homojunction, the Vienna Ab initio Simulation Package (VASP) [26] was used to perform our simulations in the framework of density-functional theory (DFT) [27]. The Perdew-Burke-Ernzerh (PBE) [28] function in the framework of Generalized Gradient Approximation (GGA) was used to describe the exchange-dependent interactions (GGA) [29]. Interlayer vdW interactions were described by using DFT-D3 function with Becke-Jonson damping [30]. To avoid the interaction between the atoms in adjacent supercells, a 20 Å vacuum layer along the z direction was set. A cut-off of 450 eV was used to explore the plane-wave calculations. Next, 9 × 9 × 1 k-mesh points [31] were used to sample the first Brillouin Zone in our total energy calculations. All atoms were relaxed completely until the force on each atom was smaller than 0.01 eV/Å. The convergence of total energy was set smaller than 10^−6^ eV. Meanwhile, the settings for band structure calculation under strain were the same as the abovementioned settings.

In order to preliminarily judge the stability of the structures with four different stacking orders, we calculated their binding energies by using Equation (1). The calculated formula of the binding energy *E*_b_ is as following [32]:

(1) $$E_{b}=E_{\alpha -\mathrm{AsP}/\gamma -\mathrm{AsP}}-\left(E_{\alpha -\mathrm{AsP}}+E_{\gamma -\mathrm{AsP}}\right)\;$$

where $E_{\alpha -\mathrm{AsP}/\gamma -\mathrm{AsP}}$ is the total energy of the α-AsP/γ-AsP homojunction, while $E_{\alpha -\mathrm{AsP}}$ and $E_{\gamma -\mathrm{AsP}}$ are energies of the α-AsP monolayer and the γ-AsP monolayer, respectively. The optical absorption coefficient $\alpha \left(\omega \right)$ [33] is presented by the formula:

(2) $$\alpha \left(\omega \right)=\sqrt{2}\omega {\left[\sqrt{\varepsilon_{1}^{2}\left(\omega \right)+\varepsilon_{2}^{2}\left(\omega \right)}-\varepsilon_{1}\left(\omega \right)\right]}^{\frac{1}{2}}\;$$

where $\varepsilon_{1}\left(\omega \right)$ and $\varepsilon_{2}\left(\omega \right)$ represent the real part and imaginary part of the dielectric function, respectively.

Energy conversion efficiency is a crucial parameter in the application of solar cells. In our work, the calculated method of PCE proposed by Scharber et al. [34] was adopted, which can be expressed as follows [34]:

(3) $$\eta =\frac{J_{SC}V_{\mathrm{OC}}\beta_{\mathrm{FF}}}{P_{\mathrm{solar}}}$$

where the short-circuit current is represented by $J_{SC}$, i.e., $J_{SC}=\int_{E_{g}^{d}}^{\infty }\frac{P\left(\hbar \omega \right)}{\hbar \omega }d\left(\hbar \omega \right)$, where $P\left(\hbar \omega \right)$ is regarded as the AM1.5 solar energy flux (presented by $\;\frac{W}{m^{2}}/\mathrm{eV}$) at the photon energy (ℏω). $E_{g}^{d}$ is the band gap of donor. $\beta_{FF}$ is the band-fill, which is a constant quantity and is often taken as 0.65. $V_{OC}$ represents the maximum open-circuit voltage, which can be expanded according to the following formula

(4) $$V_{OC}=E_{g}^{d}-\Delta E_{C}-0.3$$

where $\Delta E_{C}$ is the conduction band offset (CBO), which is the difference of the electron affinity (EA) of the two monolayers in homojunction.

## 3. Results and Discussion

### 3.1. Configurations and Stability of α-AsP/γ-AsP Homojunction

For designing the α-AsP/γ-AsP vertical homojunction, we firstly explored the monolayer structure of α-AsP and γ-AsP, respectively. The space group of the α-AsP monolayer is Pmn21. After geometry optimization, it is found that the optimized lattice constant is a = 3.50 Å and b = 4.69 Å, respectively. The γ-AsP monolayer has the same space group of Pmn21. However, the lattice constant in its unit cell is a = 3.44 Å and b = 5.65 Å, respectively. Our above calculated results on the α-AsP monolayer and γ-AsP monolayer are consistent with previous reports [16]. As reported in the study of heterojunctions [15], lattice mismatch existed after the construction of a homojunction. For our proposed vertical homojunction of α-AsP/γ-AsP, it can be found that the lattice mismatch along the x-direction can be negligible. However, there is a slight squeeze in the monolayer of γ-AsP induced by the slight mismatch along the y-direction.

**Figure 1:**
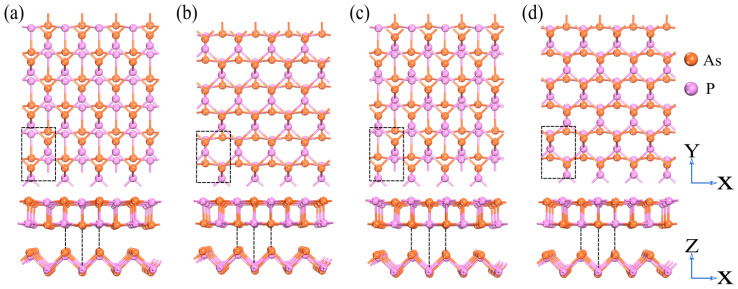
Four type of stacking configurations of the α-AsP/γ-AsP homojunction: (**a**) AA-stacking, (**b**) AB-stacking, (**c**) AC-stacking, and (**d**) AD-stacking. The orange and purple balls in the picture represent As and P atoms, respectively.

Four different stacking configurations of the α-AsP/γ-AsP homojunction are proposed. The top and side view of AA-, AB-, AC-, and AD-stacking are indicated in Figure 1a–d, respectively. The configuration of AA-stacking can be looked at as a α-AsP monolayer stacking on the bottom γ-AsP monolayer. The configuration of AB-stacking is represented by translating half one unit cell of the upper layer of α-AsP along the x-axis on the basis of the AA-stacking. The AC-stacking is tantamount to vertically inversion of the α-AsP monolayer in AA-stacking by $\;180^{{}^{\circ}}$. Correspondingly, the structure of AD-stacking can be regarded as the upper layer of α-AsP moving half one unit cell along the x-axis on the basis of the AC-stacking. By comparing the binding energies as listed in Table 1, it is found that the most stable configuration is the AA-stacking. By using molecular dynamics (MD) simulation, we checked the thermal dynamics stability of AA-stacking. As shown in Figure S1 in Supplementary Materials, the energy of the studied configuration of AA-stacking has only a small fluctuation after 10,000 fs MD running (300K). Moreover, we calculated the phonon dispersion of the α-AsP monolayer, γ-AsP monolayer, and α-AsP/γ-AsP homojunction, respectively. As shown in Figure S4 in Supplementary Materials, the calculated results show that the above three structures are stable due to no imaginary frequency existing. It implies that the α-AsP/γ-AsP homojunction of AA-stacking is preferable. Based on above analysis, we perform further study on the basis of the configuration of AA-stacking. As listed in Table 1, its optimized lattice constant in the stacking unit cell is a = 3.41 Å and b = 5.31 Å, respectively.

### 3.2. Band Structure and Density of States of α-AsP/γ-AsP Homojunction

**Figure 2:**
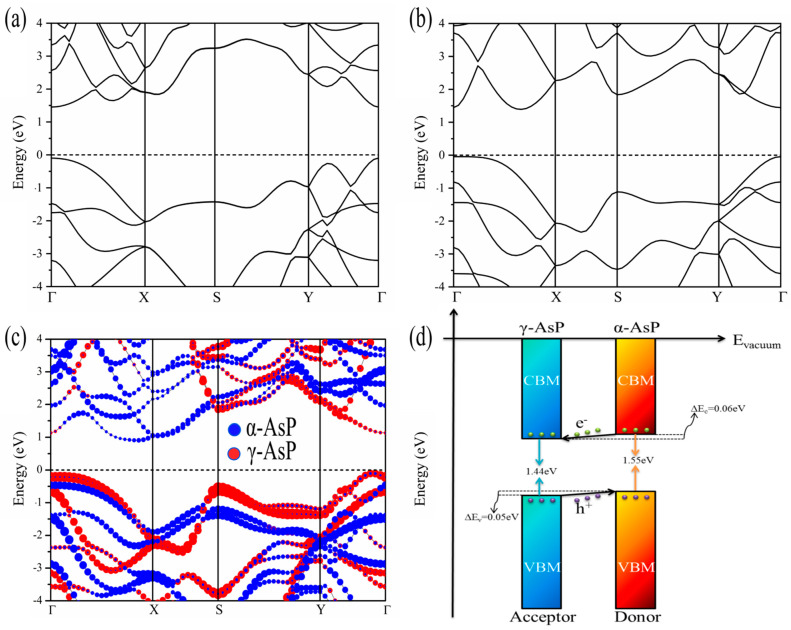
The band structure of the α-AsP monolayer (**a**) and γ-AsP monolayer (**b**). (**c**) Projected band structure of the α-AsP/γ-AsP homojunction. The blue and red circle size reflects the weight of α-AsP and γ-AsP in the band structure of the α-AsP/γ-AsP homojunction. (**d**) Band alignment of the α-AsP/γ-AsPhomojunction. The above calculations are based on the calculations of HSE06 function.

The band structures of the α-AsP monolayer, γ-AsP monolayer, and α-AsP/γ-AsP homojunction obtained from HSE06 calculations are shown in Figure 2. As indicated in Figure 2a, the α-AsP monolayer exhibits a direct band gap of 1.55 eV. In contrast, the γ-AsP monolayer has an indirect band gap of 1.44 eV, as Figure 2b indicated. It is noted that our calculated band gap of α-AsP and γ-AsP align well with previous reports [16]. In Figure 2c, it can be found that the α-AsP/γ-AsP vertical homojunction possesses an indirect band gap of 1.01 eV. We found that the CBM of the band structure in the homojunction is mainly contributed by the α-AsP monolayer, while the VBM is composed of the γ-AsP monolayer. As indicated by the lines in red and blue in Figure 2c, the degree of electron contribution from α-AsP and γ-AsP can be distinguished from each other. Detailed analysis indicates that for CBM, 71% of electrons are provided by α-AsP, while the remaining 29% of the electrons are originated from γ-AsP. For VBM, it was found that about 66% of the electrons are originated by the γ-AsP monolayer, while the remaining electrons are provided by the α-AsP monolayer. As Figure 2d indicated, our designed α-AsP/γ-AsP vertical homojunction exhibits a type-II band alignment. This kind of band alignment can promote the separation between holes and electrons in the recombination of electron-hole pairs. It can be also found that the donor band gap $\left(E_{g}^{d}\right)$ is 1.55 eV.

**Figure 3:**
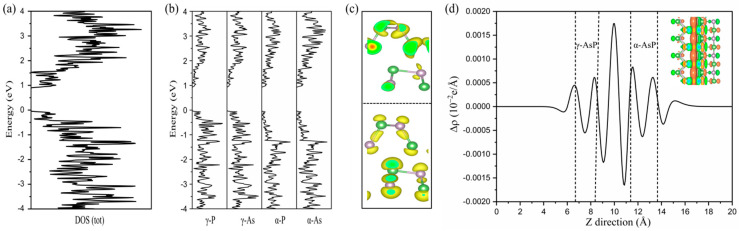
Density of states (DOS) (**a**) and projected density of states (PDOS) of the α-AsP/γ-AsP homojunction (**b**). (**c**) Partial charge density of CBM and VBM. (**d**) The plane averaged differential charge density. The isosurface in green and orange represents the loss and gain of electrons, respectively. The above calculations are based on the calculations of HSE06 function.

The work function (WF) of each component layer in the α-AsP/γ-AsP homojunction is a critical factor for the band alignment. The work function is calculated by using the formula $\;\Phi =E_{vac}-E_{F}$, where $E_{vac}$ is the vacuum level. $E_{F}$ is the Fermi level of the studied homojunction. By our calculations, the work functions of the α-AsP monolayer, γ-AsP monolayer, and α-AsP/γ-AsP homojunction are 4.81 eV, 4.82 eV, and 4.71 eV, respectively. It implies that the α-AsP/γ-AsP homojunction has a similar electron-binding capacity with its components. By analysing Figure 3d, it implies charge is transferred from the α-AsP monolayer to the γ-AsP monolayer. In contrast, by Bader charge analysis, it is found that only 0.03 e is transferred from the γ-AsP layer to the α-AsP layer. The valence band offset (VBO) in the band structure of the α-AsP/γ-AsP homojunction is 0.05 eV, while the CBO is 0.06 eV. It means that electrons can be transferred from the valence band to the bottom of the conduction band if photons are irradiated in the homojunction. In addition, the type-II band alignment of homojunction can promote the photo-generated electrons in the α-AsP to γ-AsP layer. It is helpful to the formation of excitons, which will prolong their lifetime [19].

### 3.3. Optical Properties

**Figure 4:**
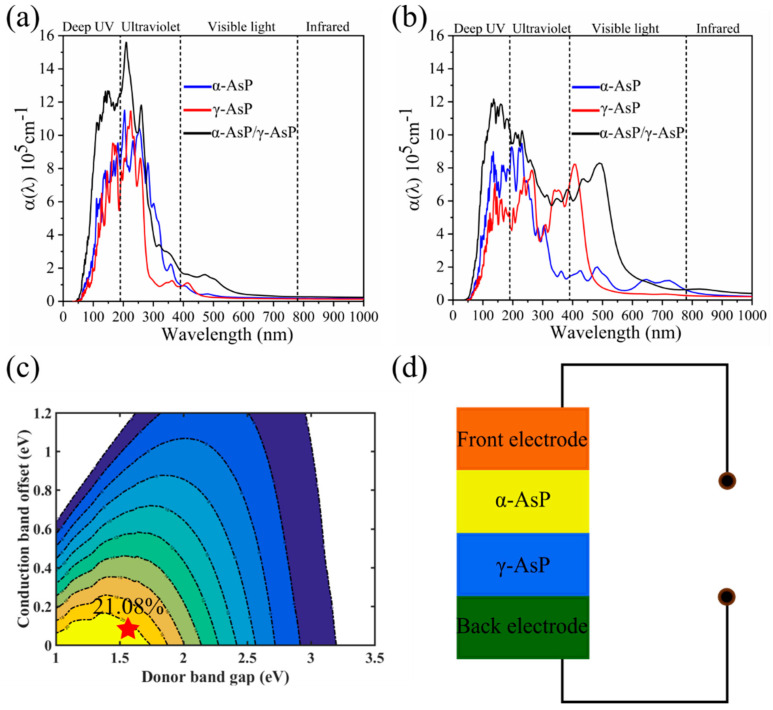
The optical absorption coefficient α of the α-AsP/γ-AsP homojunction in (**a**) zigzag direction and (**b**) armchair direction. (**c**) The computed PCE (%) of α-AsP/γ-AsP homojunction is indicated by the red star. (**d**) Schematic diagram of a solar thin-film cell. (The above calculations are based on HSE06).

Previous studies have shown that the α-AsP monolayer is a good solar cell material due to its suitable band gap as well as high electron mobility [16]. To explore the optical properties of the α-AsP/γ-AsP homojunction, the optical absorption coefficient α(ω) of the α-AsP/γ-AsP homojunction ia discussed. In Figure 4a,b, the optical absorption coefficient α(ω) along the zigzag and armchair direction is displayed. It is found that the optical absorption coefficient α(ω) of the α-AsP/γ-AsP homojunction is anisotropy. The optical adsorption of the α-AsP/γ-AsP homojunction exhibits a broad range from ultraviolet to infrared wavelength with a high order of 10^5^. The optical absorption intensity we found in the α-AsP/γ-AsP homojunction is higher than that found in GeSe/SnSe [20] and GeSe/SnS [35] heterostructures. Especially in the ultraviolet region, the peak of the optical adsorption along both directions actually reaches an order of 10^6^. This high absorptivity and anisotropy from ultraviolet to infrared implies that the α-AsP/γ-AsP homostructure can be used in polarized optical sensors and photodetectors. Considering ${\Delta E}_{C}=0.06\;\mathrm{eV}$ and $E_{g}^{d}=1.55\;\mathrm{eV}$, we can obtain the PCE from Formula (3). As shown in Figure 4c, the predicted PCE of α-AsP/γ-AsP homojunction has a high value of 21.08%. The achieved PCE in α-AsP/γ-AsP homojunction is higher than that in other hetero-structure solar cells, such as bilayer phosphorus/MoS_2_ (18%) [36], and GeSe/SnS (18%) [35] bilayer heterostructures.

### 3.4. Effect of Strain

**Figure 5:**
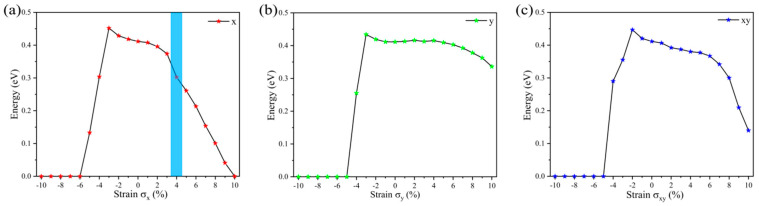
Band gap along with the applied in-plane strain σ in the α-AsP/γ-AsPhomojunction, with (**a**) strain along x (zigzag) direction, (**b**) strain along y (armchair) direction, and (**c**) biaxial strain.

In our simulation, both a uniaxial strain and biaxial strain are applied to explore the strain effect in a α-AsP/γ-AsP homojunction. As previously reported, strain can be looked at as an elastic field applied to the material [37]. By utilizing in-plane strain in the α-AsP/γ-AsP homojunction, the obtained results of the band gap are shown in Figure 5. It is found that the band gap decreases along with the increasing of the tensile strain; specifically, a transition of the band gap from indirect to direct is found under 4% tensile strain. It can be found that the band gap exhibits a slight increasing when the compressive strain along the x-axis is applied from 0% to 3%. When the compressed strain is applied in a range from 3% to 6%, the band gap is significantly decreased; specifically, if a compressed strain of 6% is applied, a transition from semiconductor to metal occurs. This is related to the change in the interlayer van der Waals forces under a compressive strain of 6%; we noticed the interlayer distance increased from 2.7 Å to 2.77 Å. Moreover, the P-As bond length was reduced from 2.36 Å to 2.31 Å in the upper layer, which caused the electron orbital reorganization and hybridization to be induced.

The band gap increases slightly if tensile strain is applied along the y-axis in a range from 0% to 2%. Then, the band-gap starts to decrease regularly. It has nearly the same trend as the compression strain along the x-axis under compression strain. The band-gap is increased when the applied compressive strain changes from 1% to 2% under biaxial strain, and then decreases sharply. The electronic property of the α-AsP/γ-AsP homojunction, in particular, changes from semiconductor to metal when the compressive strain reaches 5%. However, the band gap decreases from 1% to 10% under tensile strain.

**Figure 6:**
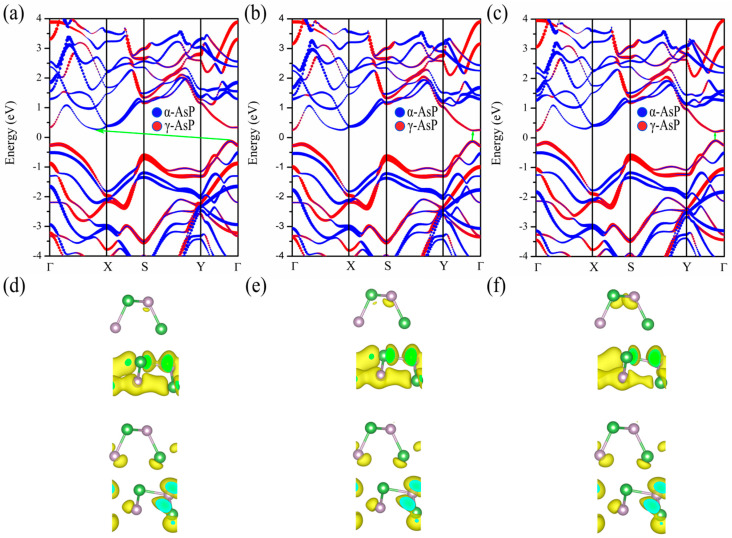
Computed band structures of the α-AsP/γ-AsP homojunction under the applied strain along x of (**a**) 2%, (**b**) 3%, and (**c**) 4%. The corresponding charge density of the CBM and VBM is shown in (**d**–**f**), respectively. The value of the isosurface is 1 × 10^−3^ e/Å^3^.

In order to fully understand the changes in electronic characteristics under different strains, we show the band structure of the α-AsP/γ-AsP homojunction under three particular tensile strain of 2%, 3%, and 4% along the x-axis in Figure 6a–c. We noted that all the projected band structures of the configurations under strains are based on PBE calculations. Under the applied tensile strain of 2% (Figure 6a), it is found that the VBM and the CBM are mainly contributed by γ-AsP and α-AsP, respectively. The charge distribution in CBM and VBM is almost completely separated under the applied strain, which implies that the α-AsP/γ-AsP homojunction is suitable for efficient solar cells. It is shown that the band alignment is same with that in its pristine structure without strain. It can be inferred that electrons and holes are perfectly separated. As shown in Figure 6c, under the strain of 4%, the α-AsP/γ-AsP homojunction exhibits a direct band-gap of 0.3 eV. In addition, the type-II band alignment in the band gap of configuration changes to type-I. It can be inferred that the VBM and the CBM are both mainly contributed by γ-AsP. As shown in Figure 6d–f, there is almost no change in the partial charge density of CBM and VBM with the increasing strain. It can be found that the charge density in the CBM is relatively dispersed, so the CBM are delocalized states. In contrast, it can be seen that the VBM exhibits strong charge density aggregation. It exhibits that VBM are localized states. Furthermore, it can be found that almost all contributions from CBM and VBM are originated from the lower γ-AsP layer. It means that the CBM and VBM are derived from the delocalized states and localized states of the γ-AsP layer, respectively. This demonstrates representative type-I band alignment. Previous study by Shang et al. [38] showed that the InSe/InTe heterostructure exhibits the characteristics of type-I band alignment, which can be used in the field of luminescent device. Our study implies that the type-II band alignment in α-AsP/γ-AsP homojunctions can be converted to type-I under tensile strain.

## 4. Conclusions

There are defects in the materials that originate from the experimental preparation. For example, the inherent defects of Bi_2_S_3_ solar cells can degrade their performance in practical applications [39]. However, Ran et al. [40] investigated the formation mechanism of defects to improve the performance of solar cells. It has been reported that the optoelectronic properties of heterojunctions can be enhanced by a sol-gel route [41] and appropriate doping [42]. It is found that the device fabricated by GaAs/AlGaAs [43] heterojunction exhibits excellent photovoltaic properties. In our optimized configurations of α-AsP/γ-AsP homojunction, the mismatch existed. We hope further study on experimental fabrication of the α-AsP/γ-AsP homojunction can consider the abovementioned factors as reported in the literature. In addition, we proposed a two-probe device model to explore the transmission probability of the α-AsP/γ-AsP homojunction. As shown in Supplementary Material (Figures S6–S8), it is found that the α-AsP/γ-AsP homojunction can generate current at lower voltages than the α-AsP monolayer and γ-AsP monolayer. More importantly, the current in the α-AsP/γ-AsP homojunction is much higher in both x and y directions than the α-AsP monolayer and the γ-AsP monolayer at the same bias voltage. It implies that the designed α-AsP/γ-AsP homojunction has potential applications in electronic nanodevices.

In summary, we study the stability, electronic structure, and optical properties of the α-AsP/γ-AsP homojunction based on vdW-corrected DFT. By estimating the binding energy, we proposed that the most stable homogeneous structure of the α-AsP/γ-AsP homojunction is AA stacking among different stacking structures. Further study on molecular dynamics simulation and phonon dispersion of the α-AsP/γ-AsP homojunction with AA stacking shows the dynamic stability of the homojunction. The type-II band alignment of the α-AsP/γ-AsP homojunction implies the perfect separation of the electron-hole pair. We also found the optical absorption coefficient of the α-AsP/γ-AsP homojunction has enhancement when compared with that in its two monolayer structures. Moreover, the absorption range can be found not only in the visible light region, but also in the ultraviolet and infrared regions. The predicted PCE of the α-AsP/γ-AsP homojunction is high at 21.08%. Interestingly, we find that when 4% uniaxial strain is applied to the α-AsP/γ-AsP homojunction, the band-gap can be tuned from indirect to direct. Our study implies that the proposed α-AsP/γ-AsP homojunction with strong light absorption coefficient and high PCE has potential application in photovoltaic devices.

## Figures and Tables

**Table 1:** The lattice constant a (Å) and b (Å), interlayer distance d (Å), and binding energy E_b_ (eV) of α-AsP/γ-AsP homojunction with four types of stacking configurations.

	a (Å)	b (Å)	d (Å)	E_b_ (eV)
AA	3.41	5.31	2.70	−2.03
AB	3.41	5.29	2.75	−2.01
AC	3.40	5.32	3.02	−1.97
AD	3.40	5.31	3.03	−1.95

## Data Availability

The data that support the findings of this study are available from the corresponding author upon reasonable request.

## Supplementary Materials

The following supporting information can be downloaded at: https://www.mdpi.com/article/10.3390/nano12101662/s1, Section S1: Thermal stability of α-AsP/γ-AsP homojunction with AA stacking; Figure S1: The molecular dynamics simulation of AA-stacking (300K); Figure S2: Comparison diagram of α-AsP/γ-AsP homojunction in initio stage and ending stage simulated by molecular dynamics; Figure S3: The molecular dynamics simulation of AA-stacking without and with strain; Section S2: Phonon dispersion of monolayer α-AsP, monolayer γ-AsP and α-AsP/γ-AsP homojunction; Figure S4: Phonon dispersion of α-AsP/γ-AsP homojunction and its components; Section S3: The comparison of band gaps obtained from PBE and HSE06 calculations; Figure S5: Comparison of band gap obtained from PBE calculation and HSE06 functional; Section S4: The I-V curve of monolayer α-AsP, monolayer γ-AsP and α-AsP/γ-AsP homojunction [44,45,46,47,48]; Figure S6: The two-probe model of transport in α-AsP/γ-AsP homojunction; Figure S7: The band structure obtained from SIESTA calculations; Figure S8: The transmission spectrum ofα-AsP/γ-AsP homojunction and its components.
