# Supplementary material for: Computational Design of α-AsP/γ-AsP Vertical Two-Dimensional Homojunction for Photovoltaic Applications
Source: Nanomaterials (Basel). 2022 May 13;12(10):1662. doi: 10.3390/nano12101662 (PMC9145734; doi:10.3390/nano12101662)
Supplement: Supplementary file 1 [file nanomaterials-12-01662-s001.zip › nanomaterials-1686086-supplementary.pdf]

Supplemental Materials

Computational Design of α-AsP/γ-AsP Vertical Two-Dimensional Homojunction for Photovoltaic Applications

Yuliang Mao [1,2,*], Yuting Du [1], Zhipeng Huang [1], Guanhua Zhang [1] and Jianmei Yuan [2,3,*]

[1] Hunan Key Laboratory for Micro-Nano Energy Materials and Devices, School of Physics and Optoelectronics, Xiangtan University, Xiangtan 411105, China;
[2] Hunan Key Laboratory for Computation and Simulation in Science and Engineering, School of Mathematics and Computational Science, Xiangtan University, Xiangtan 411105, China
[3] Hunan National Center for Applied Mathematics, Xiangtan 411105, China
* Correspondence: ylmao@xtu.edu.cn (Y.M.); yuanjm@xtu.edu.cn (J.Y.)

Section S1: Thermal stability of α-AsP/γ-AsP homojunction with AA stacking

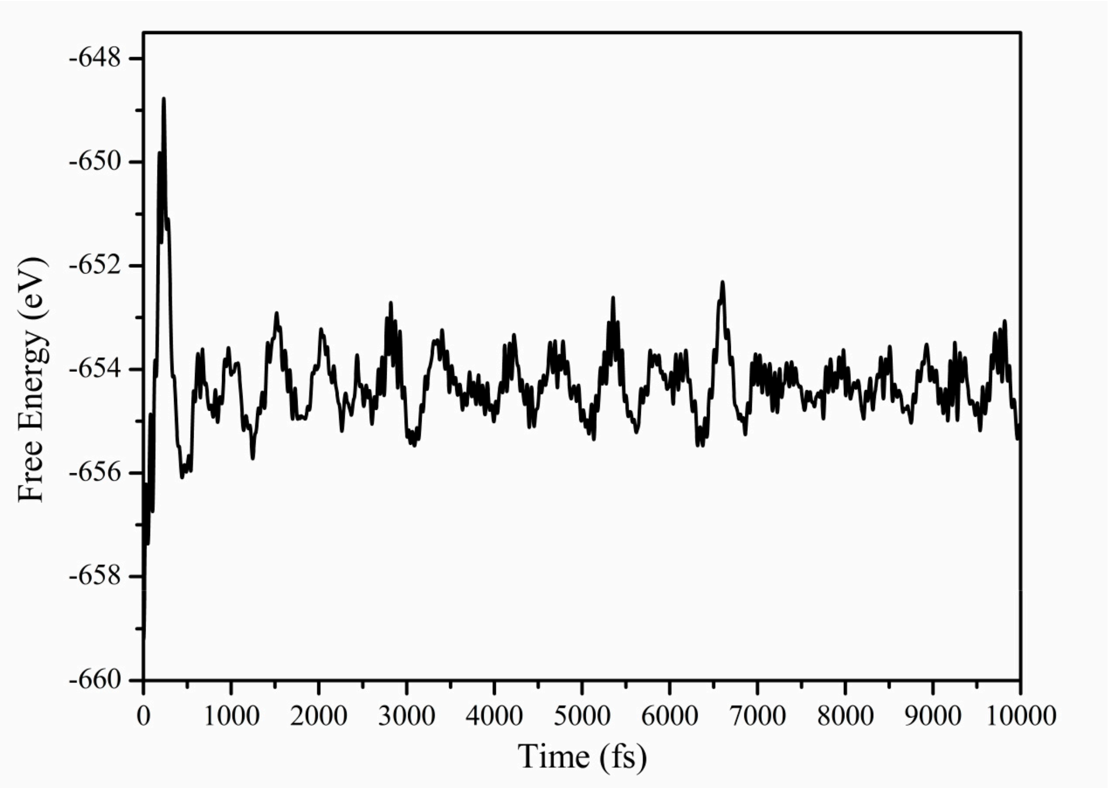

Figure. S1 The molecular dynamics simulation of AA-stacking (300K). We used 10,000 fs of molecular dynamics simulation to verify its dynamic stability.

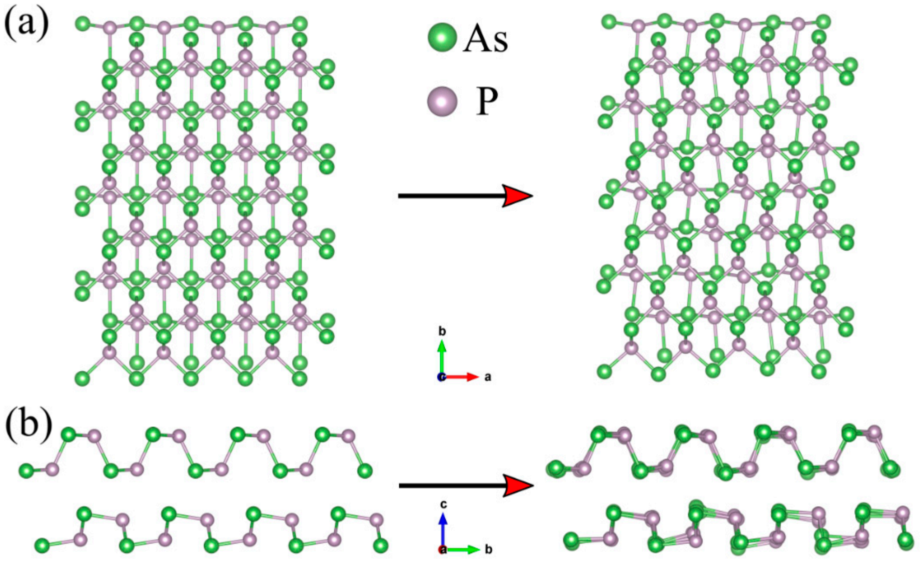

Figure. S2 Comparison diagram of α-AsP/γ-AsP homojunction in initio stage and ending stage simulated by molecular dynamics. (**a**) Top view and (**b**) side view, respectively.

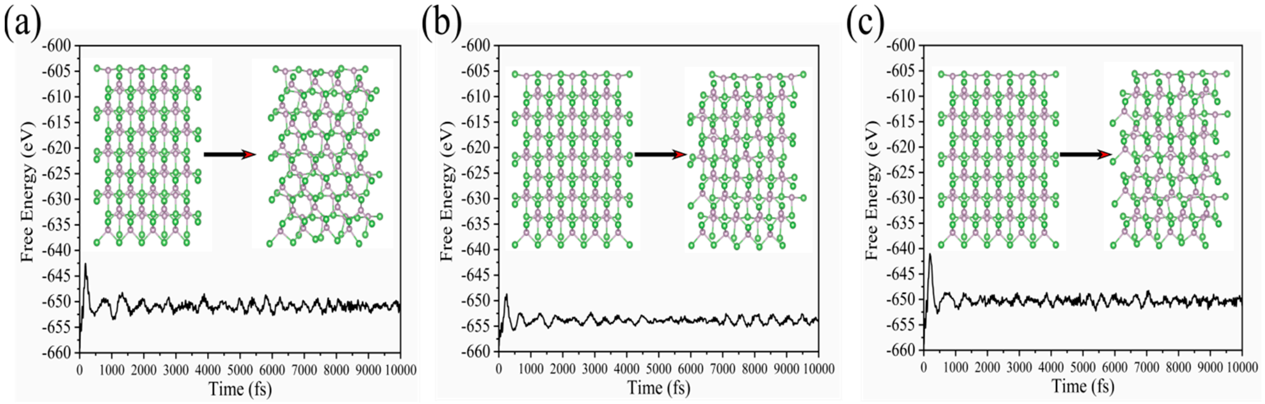

Figure. S3 (**a**) The molecular dynamics simulation of AA-stacking (500K). The molecular dynamics simulations at 300k (**b**) and 500k (**c**) with 4% tensile strain along the x-direction, respectively. The insets are top-view of α-AsP/γ-AsP homojunction in initio stage and ending stage simulated by molecular dynamics.

It can be found from Figure S1 that the free energy fluctuates in a narrow range (-655 eV~-653 eV). In Figure S2, by comparing the initial structure with the structure after molecular dynamics simulation, we can find that the structure is very stable.

Figure S3 shows that the homojunction at 500k is also relatively stable. Moreover, the homojunction under tensile strain remains stable under molecular dynamics simulations (300k and 500k).

Seciton S2: Phonon dispersion of monolayer α-AsP, monolayer γ-AsP and α-AsP/γ-AsP homojunction

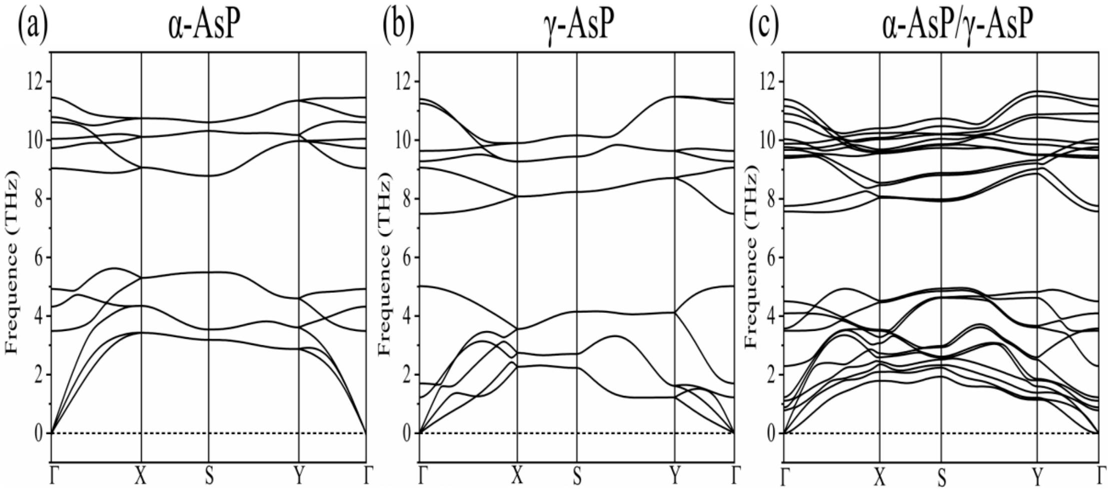

Figure. S4 Phonon dispersion of (a) monolayer α-AsP, (b) monolayer γ-AsP and (c) α-AsP/γ-AsP homojunction.

To verify the dynamic stability of the material, we calculated the phonon spectra of monolayer α-AsP, monolayer γ-AsP and α-AsP/γ-AsP homojunction, respectively. The calculated results show that above three structures are stable due to no imaginary frequency is existed, which indicates that their dynamic characteristics are very stable.

Secition S3: The comparison of band gaps obtained from PBE and HSE06 calculations.

As shown in Figure. S5, it can be found that the calculated band gap of HSE06 is about 31% larger than that obtained from the PBE functional. However, we found that the variation trend of the band gap calculated from two functionals is consistent. Considering the high time-consuming in HSE06 calculation, we adopted the PBE functional for our calculations.

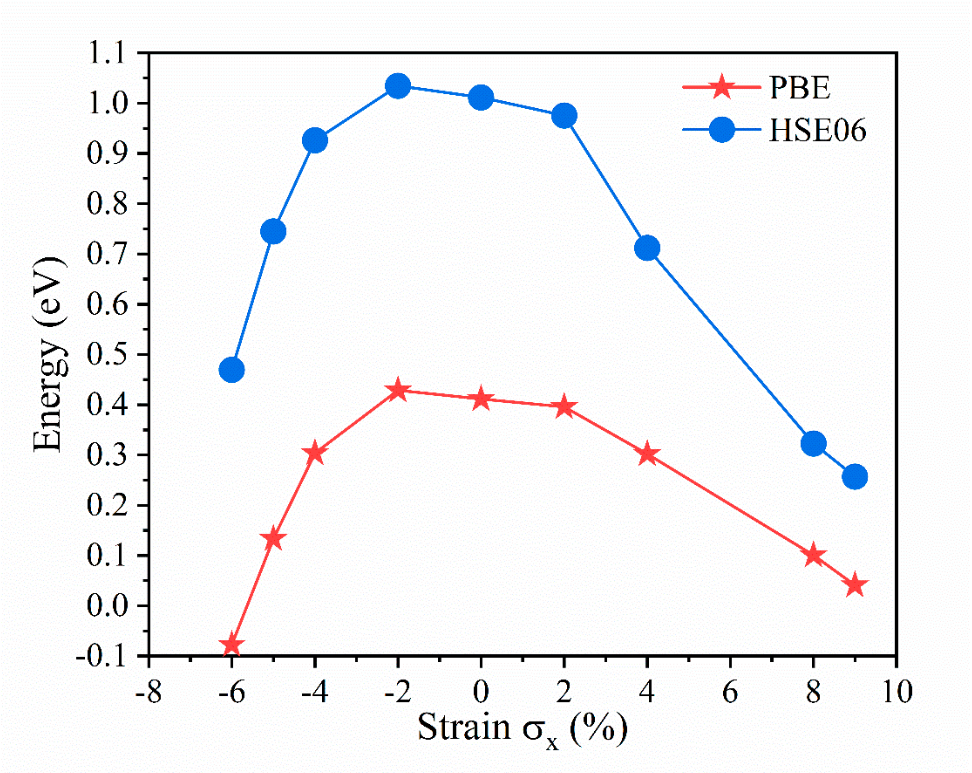

Figure. S5 Comparison of band gap obtained from PBE calculation and HSE06 functional

Secition S4: The I-V curve of monolayer α-AsP, monolayer γ-AsP and α-AsP/γ-AsP homojunction

The electron transport properties of α-AsP/γ-AsP homojunction are calculated using the TRANSIESTA module based on the non-equilibrium Green's function (NEGF) method [1] in the SIESTA package [2]. As shown in Figure. S6, we first designed a dual probe model, which consists of left and right electrodes and a central scattering region. When a bias voltage is applied to the dual probe model, we calculate the current through the node by the following method. According to Landauer–Büttiker formula [3]:

$$I(V) = \frac{2e}{h} \int T(E, V)[f(E - \mu_L) - f(E - \mu_R)]dE \qquad \text{(Equation S1)}$$

In this formula, f is the Fermi distribution function, $\mu_{L/R}$ is the chemical potential of the left and right electrodes, and an electron with energy E, under the condition of bias voltage V, the transmission coefficient from the left electrode to the right electrode is $T(E, V)$. When no external voltage is present, the equilibrium conductance of the system can be expressed as:

$$G = \frac{2e^2}{h} T(E_F, V = 0\ V) \qquad \text{(Equation S2)}$$

In our simulation, the Perdew-Burke-Ernzerh (PBE) function in the framework of Generalized-Gradient-Approximation (GGA) was used to describe the exchange-dependent interactions (GGA) [4]. For homogeneous junctions, we use the van der Waals Berland-Per Hyldgaard (BH) exchange-correlation functionals to describe van der Waals interactions [5]. All calculations were performed using the double-z-polarized (DZP) basis set with an energy shift of 100 meV and a suitable mesh cutoff is set to 300 Ry. All atoms are relaxed completely until the force on each atom was smaller than 0.01 eV/Å. The convergence of total energy was set smaller than 10^{-5} eV. For α-AsP, γ-AsP and α-AsP/γ-AsP, the k-point is set to 50×9 ×1, when calculating the transport properties along the x-axis, while a k mesh of 9×50×1 is used when calculating the transport properties along the y-axis.

To ensure the accuracy of the calculation, we first calculated the band structure of the monolayer α-AsP, the monolayer γ-AsP and the α-AsP/γ-AsP homojunction using the SIESTA software. In the band structure calculation, a k-mesh of 9×9×1 is used to sample the first Brillouin zone, and the energy cutoff is set to 300 Ry. The energy band structure is shown in the Figure. S7, it can be found that the monolayer α-AsP exhibits a direct band gap of 0.96 eV (0.90 eV). In contrast,

the monolayer γ-AsP has an indirect band gap of 0.84 eV (0.77 eV). It can also be found that the α-Asp/γ-Asp vertical homogenous junction has an indirect band gap of 0.43 eV (0.41 eV). These results are quite consistent with those calculated by VASP using PBE exchange correlation function.

We calculated the transmission spectra of the monolayer α-AsP, the monolayer γ-AsP and the α-AsP/γ-AsP homojunction along the x-axis direction and y-axis direction with zero bias. We then calculate the I-V characteristic curve at bias voltage.

The transmission gaps of the α-AsP/γ-AsP homojunction along the x and y directions are almost the same under zero bias. In Figure. S8(a) and Figure. S8(b)，it can been found that the probability of electron transmission near Fermi's surface under zero bias is zero. The transmission gap of the α-AsP/γ-AsP homojunction is narrower than the monolayer α-AsP and the monolayer γ-AsP, both along the x-axis and the y-axis. In addition, the transmission probability of the α-AsP/γ-AsP homojunction is also improved compared to the two monolayer structures.

In Figure. S8(c) and Figure. S8(d), we show the I-V characteristic curve of the monolayer α-AsP, the monolayer γ-AsP and the α-AsP/γ-AsP homojunction. At small bias voltages, the α-AsP/γ-AsP homojunction produces no current along the x- and y-axes. Both directions of the α-AsP/γ-AsP homojunction show semiconducting I-V characteristic curves, and the current nonlinearity increases rapidly after conduction. Meanwhile, we found that the α-AsP/γ-AsP homojunction can generate current at lower voltages than the monolayer α-AsP and the monolayer γ-AsP. More importantly, the current in the α-AsP/γ-AsP homojunction is much higher in both directions than the monolayer α-AsP and the monolayer γ-AsP at the same bias voltage.

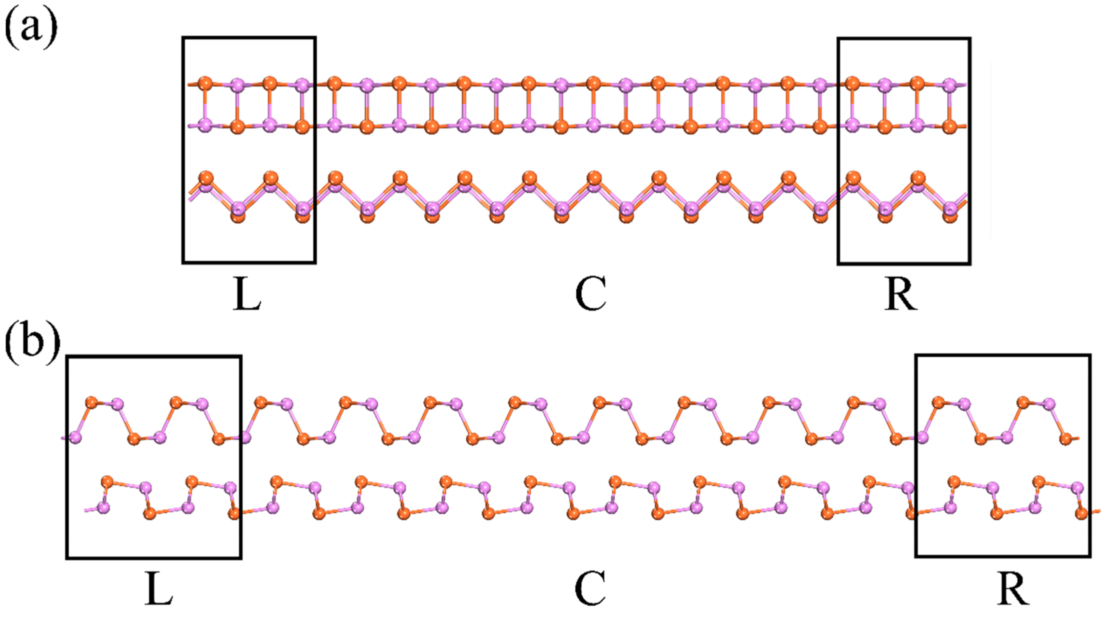

Figure. S6 The two-probe model of transport in α-AsP/γ-AsP homojunction. The two-probe model is divided into three regions: the left electrode (L), the central scattering region (C), and the right electrode (R). Schematic diagram of the structure along the x-direction (**a**) and along the y-direction (**b**).

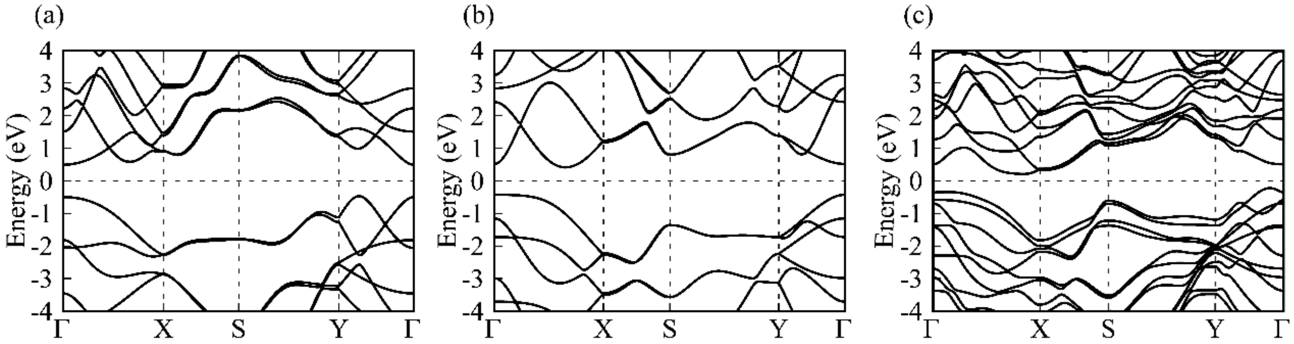

Figure. S7 The band structure obtained from SIESTA calculations. (**a**) α-AsP monolayer, (**b**) γ-AsP monolayer, and (**c**) α-AsP/γ-AsP homojunction.

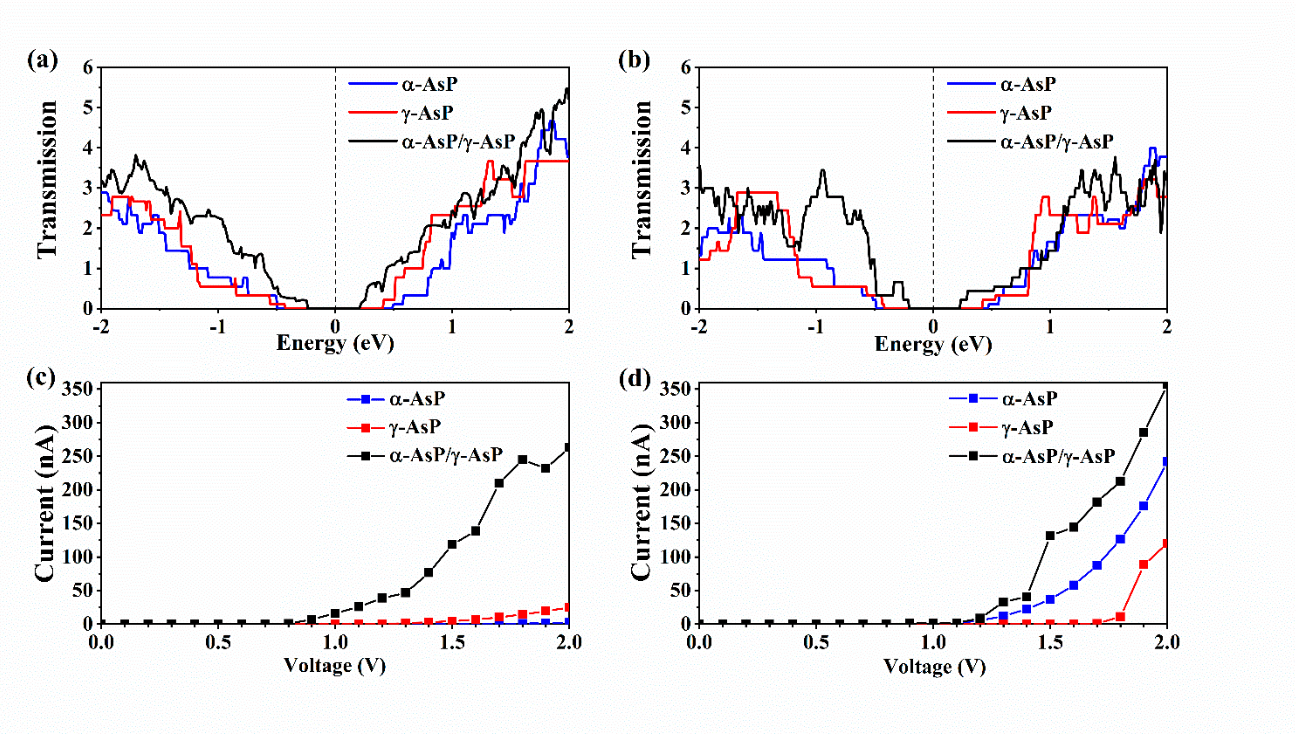

Figure. S8 The transmission spectrum of the monolayer α-AsP, the monolayer γ-AsP and the α-AsP/γ-AsP homojunction along the x-axis (a) and along the y-axis (b); The I-V curve of monolayer α-AsP, monolayer γ-AsP and α-AsP/γ-AsP homojunction along the x-axis (c) and along the y-axis (d).

References

[1] M. Brandbyge, J.-L. Mozos, P. Ordejón, J. Taylor, K. Stokbro, Density-functional method for nonequilibrium electron transport, Physical Review B, 65 (2002) 165401.

[2] J.M. Soler, E. Artacho, J.D. Gale, A. García, J. Junquera, P. Ordejón, D. Sánchez-Portal, The SIESTA method for ab initio order-N materials simulation, Journal of Physics: Condensed Matter, 14 (2002) 2745.

[3] M. Büttiker, Y. Imry, R. Landauer, S. Pinhas, Generalized many-channel conductance formula with application to small rings, Physical Review B, 31 (1985) 6207.

[4] J.P. Perdew, K. Burke, M. Ernzerhof, Generalized gradient approximation made simple, Physical review letters, 77 (1996) 3865.

[5] I. Hamada, van der Waals density functional made accurate, Physical Review B, 89 (2014) 121103.
